# One Year After Mild COVID-19: Emotional Distress but Preserved Cognition in Healthcare Workers

**DOI:** 10.3390/jcm14176007

**Published:** 2025-08-25

**Authors:** Irene Peláez, David Martínez-Íñigo, Roberto Fernandes-Magalhaes, María E. De Lahoz, Ana Belén del Pino, Sonia Pérez-Aranda, Alejandro García-Romero, Dino Soldic, Francisco Mercado

**Affiliations:** 1 Department of Psychology, School of Health Sciences, Rey Juan Carlos University, 28922 Madrid, Spain; david.martinez@urjc.es (D.M.-Í.); roberto.fernandes@urjc.es (R.F.-M.); mariaeugenia.delahoz@urjc.es (M.E.D.L.); belen.delpino@urjc.es (A.B.d.P.); alejandro.garcia@urjc.es (A.G.-R.); dino.soldic@urjc.es (D.S.); francisco.mercado@urjc.es (F.M.); 2 Research Group in Cognitive Neuroscience, Pain and Rehabilitation (NECODOR), Rey Juan Carlos University, 28922 Alcorcón, Spain; 3 Department of Psychiatry, University Hospital of Alcorcon, 28922 Madrid, Spain; spereza@fhalcorcon.es

**Keywords:** cognition, healthcare, long-term effects, mild COVID-19, neuropsychology

## Abstract

**Background/Objectives:** Although COVID-19 may cause cognitive impairments for up to six months, the long-term effects of mild cases remain unclear. Given their high exposure and critical role in public health, assessing this impact on healthcare workers is essential. Aim: The present study aimed to examine the cognitive and emotional effects of mild COVID-19 in 92 healthcare workers one year after infection. **Methods**: In total, 50 had experienced mild COVID-19, while 42 had not been infected. Participants completed a neuropsychological assessment evaluating attention, memory, and executive functions, along with self-reported measures of anxiety, depression, post-traumatic stress, occupational stress, and burnout. **Results**: No significant cognitive differences were observed between the groups. However, both exhibited moderate-to-severe psychological distress, with the COVID-19 group showing higher trait anxiety (*p* = 0.032). Emotional symptoms were significantly associated with neuropsychological performance—higher burnout (ρ from −0.20 to −0.28, *p* < 0.05) and stress (ρ from −0.25 to −0.33, *p* < 0.01) correlated with slower responses and more errors in tasks such as the D2 variation index, TESEN execution speed, Rey–Osterrieth Figure recall, and Digit Span forward span. **Conclusions**: These findings suggest no long-term cognitive impairment after mild COVID-19 but highlight the substantial emotional toll of the pandemic on healthcare workers. Future research should explore cognitive reserve as a protective factor.

## 1. Introduction

In the fourth year of the pandemic, substantial evidence underscores that Coronavirus Disease 2019 (COVID-19) is a systemic illness that frequently affects the central nervous system. As of November 2024, data from the World Health Organization has confirmed 776,546,006 reported infections globally, including approximately 14 million cases in Spain [1]. The global investigation of this virus is unprecedented, with numerous emerging medical studies describing symptom profiles and treatment outcomes in COVID-19. Despite the extensive literature on COVID-19 and its effects on the brain, the long-term cognitive impact of the virus remains poorly understood. Addressing these issues is particularly critical for healthcare workers, who represent a population heavily exposed to the virus.

The virus responsible for COVID-19 is severe acute respiratory syndrome coronavirus 2 (SARS-CoV-2), a member of the coronavirus (CoVs) family [2]. Common symptoms include fever, cough, headache, myalgia, respiratory and gastrointestinal issues, and metabolic and immune disturbances [3,4]. Neurological symptoms have also been described, including taste and smell dysfunction, encephalopathy, myelitis, seizures, ischemic stroke, and impaired consciousness [3]. The association between respiratory viral infections and neurological symptoms remains unclear, and various hypotheses have been proposed [5]. The most widely accepted causes include cerebrovascular factors (ischemic strokes, intracranial hemorrhage, and cerebral microbleeds), neuroinflammation, dysregulation of the immune response, and direct viral invasion of the brain via the olfactory bulb [3,4,6]. Given its connections with the rest of the central nervous system, entry through the olfactory bulb could explain some neurological and cognitive symptoms [5,6].

Previous studies have signaled that individuals with acute SARS-CoV-2 infection commonly complain about cognitive impairments [7]. This often has been described as ‘brain fog’ [8]. The prevalence and duration of self-reported cognitive symptoms post-COVID-19 are well documented, although estimates vary considerably across studies [5]. Beyond these subjective cognitive complaints, several studies have also documented objective cognitive impairments, including low energy, disorientation, attentional deficits, impairments in learning and memory retrieval, and executive dysfunction [3,4,9,10]. Research indicates that these symptoms are relatively common, affecting 20–36% of individuals within the first three months post-infection, with attention and memory being the most affected domains [11]. Data derived from a large body of studies suggest that the short- and medium-term cognitive effects are relatively clear, with general cognitive decline observed up to six months post-infection [12,13]. Longitudinal studies have extended previous findings showing that working memory deficits, among other cognitive alterations, are noticeable in COVID-19 patients even one year after infection [14,15].

A key point is that most of these studies have included patients with acute COVID-19 who had been previously hospitalized or were still hospitalized during the evaluation period [5,8]. Cognitive disturbances have been consistently described in patients who have undergone prolonged hospitalizations or intensive care [14,15]. Few studies have examined the effects of COVID-19 on cognition by distinguishing between participants who required hospitalization and those with milder symptoms [8,10,15]. These studies have found that cognitive deficits are significantly more pronounced in patients with severe infections than in those experiencing mild or no symptoms. Although most of the population infected during these years has experienced mild infection [16], it remains unclear whether these milder cases (i.e., those not requiring hospitalization) also cause long-term cognitive deficits that could significantly impact quality of life and emotional well-being.

Mood disturbances, such as anxiety, depression, and post-traumatic stress disorder, have also been frequently reported by patients who have suffered from COVID-19, persisting for several months after the infection [15,17,18]. These emotional disturbances have been suggested to negatively impact cognitive processes such as attention, comprehension, and decision-making [15,19]. The risk of developing such symptoms is particularly pronounced among frontline healthcare workers involved in diagnosing, treating, and caring for infected patients [17,20]. At the same time, the healthcare environment has been characterized by high levels of occupational distress, defined by the ICD-11 [21] as chronic workplace stress that has not been successfully managed, resulting in significant impairment. This includes professional burnout and moral distress, even before the COVID-19 pandemic began. The prolonged duration of this situation has increased the risk of healthcare workers developing chronic occupational stress [22,23,24,25]. For this reason, exploring the potential effects of mild COVID-19 on cognition and distinguishing them from those linked to affective symptoms is of particular importance.

With this in mind, our study had two main objectives: (1) to investigate the long-term cognitive effects of mild COVID-19 infection—characterized by brief symptoms that did not require hospitalization and reflecting the experience of the majority of the population—using a comprehensive neuropsychological battery, and (2) to explore the potential relationships between cognitive functioning and emotional status (anxiety, depression, post-traumatic stress disorder, and occupational burnout) in healthcare professionals, considering their high exposure to the virus and the considerable pressure they face daily in their occupation. While mild infections are common, their long-term impact on cognition and mental health in healthcare workers remains largely unexplored, motivating our investigation.

## 2. Materials and Methods

### 2.1. Participants

In total, 92 right-handed healthcare professionals (78 women) from the University Hospital Alcorcon Foundation (HUFA), a large hospital within the public health network of the Community of Madrid, Spain, participated in this study. This sample size is similar to previous neuropsychological studies conducted on patients affected by COVID-19 disease [7,14]. Nevertheless, as previously recommended [26], we performed a sensitivity analysis based on the sample size (N = 92) using G*Power 3.1.9.7 [27]. Using α = 0.05 and power = 0.80, it was determined that the current study was powered to detect effect sizes of f ≥ 0.29. Participants were between 22 and 61 years old. The COVID-19 group (50 participants) included professionals who had received outpatient treatment after experiencing a mild infection, defined as symptoms confined to the upper respiratory tract and/or mild systemic manifestations (e.g., fever, myalgia, fatigue) without signs of pneumonia or hypoxia and resolving within a few weeks. At the time of their participation in the study, the mean time elapsed since the acute infection was 13.26 months (SD = 3.33). The control group (42 participants) consisted of healthcare professionals who had not suffered from COVID-19, verified through bimonthly serological tests at HUFA to detect antibodies. Participants suffering from neurological diseases or disorders that impair cognitive function, psychosis, or substance abuse/dependence were excluded. The Rey Juan Carlos University Research Ethics Board (number: 2212202001021) and the ethics committee of University Hospital Alcorcon Foundation (HUFA) approved the study in accordance with their respective requirements. After obtaining written informed consent for their participation, participants provided sociodemographic data (age, level of education, profession) and information on their COVID-19 symptoms and treatment. The sociodemographic characteristics of the participants are shown in Table 1, along with information about COVID-19 symptoms (only for the COVID-19 group).

### 2.2. Neuropsychological Assessments

Participants completed a comprehensive battery of neuropsychological tests to assess attention, memory, and executive functions. Attentional status was assessed by the Spanish version of the D2 Sustained Attention Test (D2 test) [28], which assesses sustained attention, selective attention, and processing speed. The Test of the Paths (TESEN) [29], based on the Trail Making Test (TMT) [30], consists of four tests that evaluate alternating attention and flexibility. The Symbol Search and Number Key subtests of the Spanish version of the Wechsler Intelligence Scale for Adults-IV (WAIS-IV) [31] were also administered to assess processing speed.

To assess memory processing, several subtests of the Spanish version of the Wechsler Memory Scale III (WMS-III) [32,33] were applied, including Word Lists I and II and Faces I and II. Additionally, the WAIS-IV vocabulary subtest was used to evaluate semantic memory. In addition, the Rey–Osterrieth Complex Figure Test [34] was used to evaluate visuospatial construction, memory retention, and the ability to recall and reproduce complex visual information.

Finally, executive functions were assessed by the Five Digit Test [35], consisting of four experimental conditions (i.e., reading, counting, selection, and switching). Specifically, this test allowed us to assess the numerical processing and processes of attentional control, interference, and flexibility. The Digit Span, Spatial Span, and Letters–Numbers Sequencing subtests of the WMS-III were applied to assess working memory. The Similarities subtest of the WAIS-IV was administered to measure verbal reasoning and abstraction capacity. Word fluency was assessed using the FAS Test, a subtest of the Neurosensory Center Comprehensive Examination for Aphasia [36]. In addition, the Key Search and Zoo subtests of the BADS Scale (Behavioral Assessment of the Dysexecutive Syndrome) [37] were administered to assess aspects related to planning. For more information on the neuropsychological assessment, see Appendix A (Table A1).

### 2.3. Emotional Measures and Subjective Complaints

Participants’ mood and clinical variables, such as anxiety and depression, were assessed using the Patient Health Questionnaire-9 (PHQ-9) [38], the State-Trait Anxiety Inventory (STAI) [39], and the Symptom Checklist-90 (SCL-90) [40]. The SCL-90 evaluates the presence of 90 symptoms, interpreted in terms of nine primary indices (somatization, obsessions and compulsions, interpersonal sensitivity, depression, anxiety, hostility, phobic anxiety, paranoid ideation, and psychoticism) and three global indices (global severity index, positive discomfort index, and total positive symptoms). Post-traumatic stress was assessed using the Post-Traumatic Stress Checklist–Civilian Version (PCL) [41]. Finally, occupational burnout was measured using the Emotional Exhaustion Subscale of Maslach’s Burnout Inventory (MBI) [42].

Additionally, participants reported concerns about changes in their abilities. Specifically, subjective cognitive complaints were assessed using the Failures in Everyday Life Questionnaire (MFE-30) [43] and the Abbreviated Prefrontal Symptom Inventory (ISP-20) [44].

### 2.4. Procedure

The Occupational Health Service of HUFA used its internal database to identify healthcare professionals with and without a history of mild COVID-19 and contacted them by telephone to invite participation in the study. Healthcare professionals who agreed to participate were subsequently contacted by Rey Juan Carlos University to verify that they met the inclusion criteria and to schedule appointments.

The neuropsychological assessment was conducted over two sessions: the first at HUFA, and the second at the Cognitive Neuroscience Laboratory of Rey Juan Carlos University. Each session lasted approximately 1.5 h. Both centers had specially conditioned rooms, optimized for lighting, sound, and ventilation to ensure a conducive testing environment.

During the first session, participants were provided with an informed consent form and a questionnaire on COVID-19 symptoms and treatment characteristics (for the COVID group only). Next, attention, memory, and executive functions were assessed using the following battery of neuropsychological tests: Word Lists I and II, Faces I and II, Letters–Numbers subtest, and Spatial Span from WMS-III. Additionally, the Five Digit Test, the FAS Test, and the Symbol Search and Number Key tests of the WAIS-IV were administered. The second session, scheduled between two and four weeks after the first, included the following tests: the D2 test; TESEN; the digits, vocabulary, and similarities subtests of the WAIS-IV; the Rey–Osterrieth Complex Figure Test; and the Key Search and Zoo subtests. Self-reported emotional questionnaires were completed online in the interim between the two sessions.

### 2.5. Statistical Analysis

Prior to the statistical analyses, the normality of the emotional measures and neuropsychological outcomes was assessed. All dependent variables followed a normal distribution, as determined by the Kolmogorov–Smirnov test (*p* > 0.05). To examine potential differences in age and education level between participants, independent samples t-tests and chi-squared (χ^2^) tests were conducted, respectively. No significant differences were found between groups for these variables (see Table 1).

A series of one-way ANOVAs was conducted to compare neuropsychological and emotional self-report measures between the COVID-19 and control groups. Emotional variables that showed significant differences between groups were included in the analyses as covariates.

Finally, bivariate correlations were conducted to examine the relationships between emotional test scores and neuropsychological performance. A one-tailed significance level of 0.05 was applied to all statistical analyses. All analyses were performed using SPSS v. 27.0.

## 3. Results

### 3.1. Descriptive Analysis

**Figure 1:**
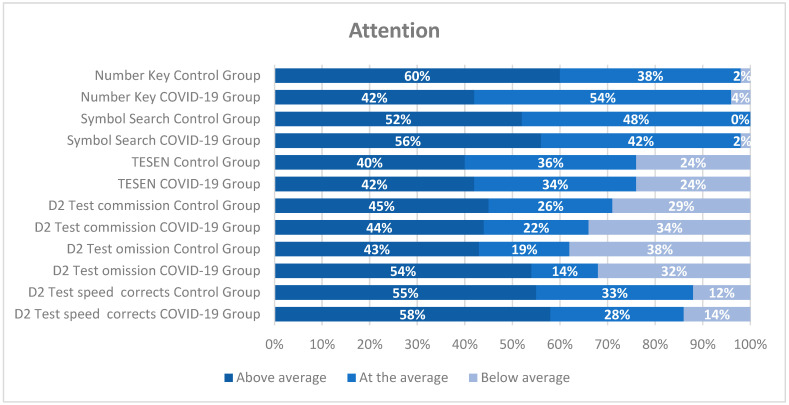
Distribution of scores on neuropsychological attention tests (in percentages) for both groups according to the intervals of each questionnaire. The classification was performed using standard scores. Abbreviations: D2 Sustained Attention Test: D2 test; Test of the Paths: TESEN.

**Figure 2:**
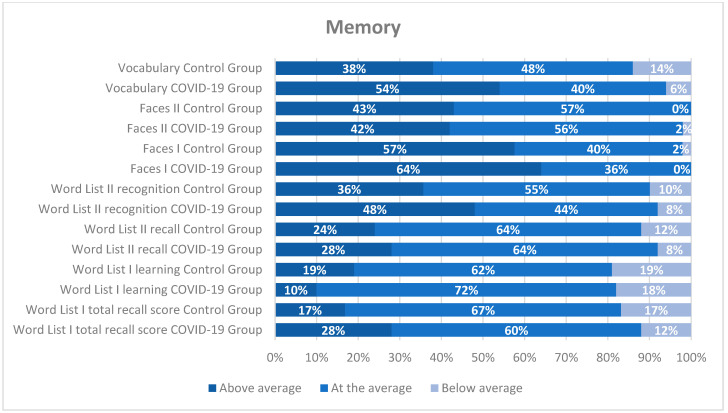
Distribution of scores on neuropsychological memory tests (in percentages) for both groups according to the intervals of each questionnaire. The classification was performed using standard scores.

**Figure 3:**
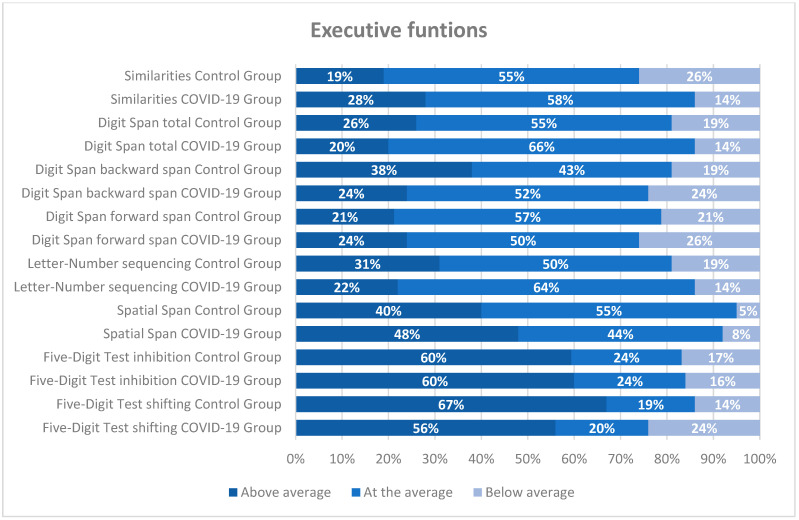
Distribution of scores on neuropsychological executive functions tests (in percentages) for both groups according to the intervals of each questionnaire. The classification was performed using standard scores.

The distribution of scores across each neuropsychological measure (see Figure 1, Figure 2 and Figure 3) was analyzed separately for the COVID-19 and control groups. In most tests, over 65% of the participants scored at or above average. However, a more detailed examination by cognitive domain is warranted. Regardless of the group, approximately 25% of participants scored below average on attention tests (TESEN and D2 test), as shown in Figure 1. A similar pattern was observed in other cognitive domains, such as executive functions (Digit Span and Five Digits), where between 14 and 24% of healthcare professionals scored below average (see Figure 3). Memory appeared to be relatively well preserved, with only about 15% or fewer healthcare workers scoring below average (see Figure 2).

**Figure 4:**
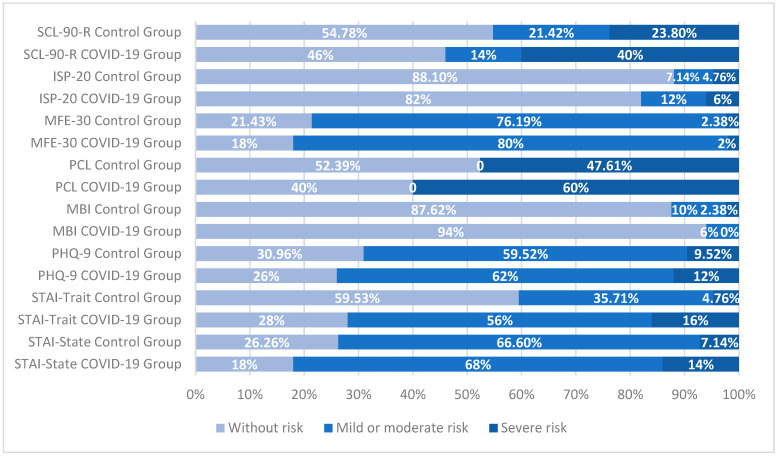
Distribution of self-reported scores (in percentage) for both groups according to the intervals of each questionnaire. Abbreviations: Symptom Checklist-90: SCL-90-R, Abbreviated Prefrontal Symptom Inventory: ISP-20, Failures in Everyday Life Questionnaire: MFE-30, Post-Traumatic Stress Checklist–Civilian Version: PCL, Emotional Exhaustion Subscale of Maslach’s Burnout Inventory: MBI, Patient Health Questionnaire-9: PHQ-9, and State-Trait Anxiety Inventory: STAI.

Regarding emotional symptoms and subjective cognitive complaints assessed through self-reported questionnaires, moderate to severe levels were observed across both groups. Specifically, participants exhibited psychological stress (49.61%), post-traumatic stress (53.80%), depression (71.52%), trait anxiety (56.23%), state anxiety (77.87%), and memory failures (80.28%). Full details on the distribution of these scores by group are shown in Figure 4.

### 3.2. Analyses of Neuropsychological Measures

The mean scores for each neuropsychological test outcome are shown in Table 2, grouped by COVID-19 and control health professionals. One-way ANOVAs comparing cognitive performance across tests revealed no significant differences between groups on any neuropsychological measure. Furthermore, all effect sizes (partial η^2^) were below the conventional threshold for a small effect (η^2^ = 0.01), indicating that any observed differences were negligible in magnitude and unlikely to have clinical relevance.

### 3.3. Analyses of Emotional Symptomatology and Subjective Complaints

The results for ANOVAs exploring potential differences between the COVID-19 and control groups in self-reported emotional scores are shown in Table 3. Although healthcare workers in the COVID-19 group generally scored higher on most emotional measures than the control group, significant differences were only observed for trait anxiety, with the COVID-19 group exhibiting higher scores than the control group [F(1.90) = 0.784, *p* = 0.032, η^2^ = 0.05]. No other statistically significant differences were found, and the corresponding effect sizes were all below the threshold for a small effect.

### 3.4. Relationship Between Neuropsychological Tests and Emotional Symptomatology

As mentioned in the data analysis section, correlations were conducted to explore potential relationships between cognitive performance, as assessed through the neuropsychological tests, and the scores from the self-reported emotional questionnaires (see Table 4). The results revealed several correlations, both positive and negative, between cognitive outcomes (such as the D2 variance index, TESEN path 4 resolution time, Rey–Osterrieth Complex Figure recovery score, and forward amplitude in the Digit Span) and most emotional symptoms. A consistent pattern emerged, where higher levels of emotional symptomatology (e.g., anxiety, depression, post-traumatic stress disorder, and work-related stress) were correlated with greater variability in cognitive performance and task resolution times, as well as reduced recall and accuracy scores.

## 4. Discussion

This study aimed to explore the impact of mild COVID-19 on cognition in healthcare professionals one year post-infection. Additionally, we examined the potential relationships between emotional symptoms—such as anxiety, depression, post-traumatic stress disorder, and work-related stress—and cognitive functioning, as these clinical factors may also influence cognitive performance in patients suffering from COVID-19 [22,45].

The results indicate that, overall, healthcare workers affected by mild COVID-19 did not show significant cognitive impairments compared with those who were not infected. Thus, both groups were comparable, with over 65% of participants in each group scoring at or above average across the different cognitive domains (attention, memory, and executive functions) assessed by a comprehensive neuropsychological battery. The cognitive effects of COVID-19 infection have been extensively documented in patients with severe infection, short recovery periods (less than 7 months), and in those involved in ‘long COVID’ research [5]. This latter term refers to a range of symptoms, including fatigue, respiratory distress, and cognitive dysfunction, which appear three months after a coronavirus infection, persist for at least two months, impact daily life, and cannot be explained by other causes [1].

Longitudinal studies are beginning to emerge, providing data on the evolution and cognitive recovery following infection in patients who required inpatient treatment [14,46]. Blazhenets and coworkers [47] found that deficits in executive functions, visuo-constructional skills, and memory persisted six months after recovering from COVID-19, as assessed by the Montreal Cognitive Assessment (MoCA). However, in most of the patients, performance significantly improved compared with the post-acute phase, suggesting that the potential effect on cognition is reversible over time. Similarly, it has been reported that 53% of patients who experienced critical symptoms of COVID-19 showed clear cognitive deficits in at least one cognitive domain within two months of recovery. However, this percentage dropped to 36% ten months after hospitalization [48]. Thus, the absence of cognitive deficits in our sample could be because the COVID-19 symptomatology was less severe (it did not require hospitalization), and the time since recovery was longer, which is reassuring for the general population.

However, alternative or complementary explanations can be considered, which, while still hypothetical and requiring further investigation, may align well with our results. One possibility is that the observed limited impact of COVID-19 on the cognitive functioning of infected healthcare workers might, at least in part, be explained by factors related to cognitive reserve, which is higher in individuals with a high level of education and occupational activity [49]. Specifically, the concept of cognitive reserve refers to a set of factors and mentally stimulating activities developed throughout life, such as social stimulation and leisure activities, which may play a protective role in brain plasticity. In this context, the cognitive demands associated with healthcare employment, combined with years of formal education (over 60% of our sample having 14 or more years), likely contribute to a cognitive status that supports optimal daily functioning [50]. Importantly, a recent systematic review and individual participant data meta-analysis by Foreman and colleagues [51] demonstrated that higher cognitive reserve significantly moderates post-COVID-19 cognitive outcomes: individuals with high cognitive reserve experienced substantially smaller cognitive deficits following infection, whereas those with low cognitive reserve showed greater impairment. Moreover, research by Panico and coworkers [52] showed that higher levels of cognitive reserve are associated with lower levels of stress, which may help mitigate cognitive impact. This is further supported by Favaretto et al. [53], who emphasized that affective temperaments and emotional factors can modulate cognitive performance, highlighting the potential interaction between emotional states and cognitive reserve in protecting cognition under stress. Conversely, Vance and coworkers [50] noted that the absence of work activities—an experience shared by the broader population confined at home during the early months of the pandemic—represents a factor that impacts cognitive reserve and, consequently, cognition. Thus, the results obtained in our sample of healthcare workers cannot be generalized to the entire population.

Regarding emotional symptomatology, self-reported measures of anxiety, depression, post-traumatic stress disorder, and occupational burnout were also collected, as these factors are known to impact cognitive performance [22,45,54]. As a result, healthcare workers in the COVID-19 group exhibited higher levels of trait anxiety than those in the control group, a measure that is generally stable over time [55], suggesting that the observed group differences may reflect pre-existing psychological traits rather than being solely attributable to COVID-19 exposure. In addition, both groups exhibited elevated emotional symptoms, with anxiety, depression, and post-traumatic stress consistently showing the highest levels. Correlation analyses further indicated that higher burnout, stress, and post-traumatic stress were modestly associated with poorer performance in cognitive tasks assessing attention (e.g., the D2 test and TESEN), executive functions (Spatial and Digit Span), and memory (Rey–Osterrieth Complex Figure). Although COVID-19 infection does not seem to be directly associated with the presence or absence of emotional disturbances, it has been suggested that prolonged exposure to the pandemic has had significant psychological effects on healthcare workers [56]. Several factors have been identified as contributing to emotional disturbances, such as working exclusively in healthcare roles, long shifts due to staff shortages, close and prolonged contact with infected patients, inadequate or insufficient use of personal protective equipment at the beginning of the pandemic, working in enclosed and crowded spaces, and poor or insufficient ventilation [20,25,55,57]. Similarly, previous research has documented a wide range of emotional health issues among healthcare workers during the pandemic, including depression, anxiety, post-traumatic stress disorder, and insomnia [18]. A recent meta-analysis [56] examined 392 studies and confirmed that between 25 and 28.5% of healthcare workers exhibited symptoms of depression, anxiety, post-traumatic stress disorder, and substance abuse. Furthermore, the prevalence of these symptoms is higher among healthcare workers than among the general population. This is unsurprising, given the increased workload, risk of infection, uncertainty, and heightened sense of threat experienced by the healthcare population during the pandemic [20,25]. However, one of the few longitudinal studies conducted so far suggests that mental health problems tended to decrease as the COVID-19 pandemic progressed [56].

Following this line of reasoning, we observed several correlations between cognitive performance and emotional symptomatology. Higher levels of psychological stress, burnout symptoms, depression, and post-traumatic stress were associated with poorer performance on the D2 variance index, TESEN path 4 resolution time, Rey–Osterrieth Complex Figure recovery score, and forward amplitude in the Digit Span Test (WAIS-IV). These findings suggest a relationship between certain cognitive processes, such as selective and sustained attention, working and visual memory, executive function, and the presence of emotional symptoms, warranting further attention. This underscores the importance of further investigation into the role emotional disturbances may play in cognitive functioning, particularly in healthcare workers—a rarely addressed topic [58]—as it allows us to distinguish the effects of COVID-19 infection from the potential impact of these emotional variables on cognition. Several studies have identified cognitive dysfunction that may persist even during the remission phase of clinically significant symptoms [45,52], particularly in processes such as executive functions, attention, working memory, and episodic memory, in individuals with depression [52], post-traumatic stress [57], burnout [22,59], and high levels of stress in general [60].

Therefore, given the absence of cognitive differences between groups (COVID-19-infected and non-infected), the high prevalence of emotional disturbances, and their correlation with cognitive performance, it is critical to prioritize monitoring the emotional well-being of health professionals. Longitudinal findings from a limited number of studies [56] indicate that pre-existing health problems have contributed to an increased risk of mental health issues during the pandemic. Therefore, it is not only crucial to continue monitoring the emotional status of health professionals but also to develop long-term strategies to address the systemic challenges they face daily.

This study is not without limitations. First, measuring neuroinflammatory levels, which have previously been shown to play a role in the development, maintenance, and recovery of cognitive and psychological symptoms [18], would have been useful for better characterizing potential cognitive impairments in the participant sample. Additionally, the lack of cognitive and psychological data prior to COVID-19 and the reliance on self-report measures for assessing emotional distress limit our ability to fully capture the impact of the disease on healthcare professionals. Self-reported data may be subject to bias, and future studies should consider incorporating objective or clinician-administered assessments to complement these measures. To better understand the long-term cognitive and emotional consequences of the pandemic, further longitudinal studies are essential. Furthermore, our sample was predominantly female and drawn from a single hospital, which limits the external validity and generalizability of our findings to broader populations, including male healthcare workers and those from diverse healthcare settings. This gender imbalance reflects the feminization of healthcare professions [61] and highlights the need for future research to include more balanced and multicenter samples to improve representativeness and applicability of the results.

## 5. Conclusions

In summary, it appears that, fortunately, the long-term effects of mild COVID-19 infection do not significantly affect the cognitive functioning of COVID-19 patients, particularly in a sample of healthcare workers. However, regardless of their COVID-19 status, healthcare workers exhibited high levels of emotional symptoms, such as post-traumatic stress, depression, and burnout, which are linked to poorer cognitive performance. The risks faced by healthcare professionals, as the first line of defense against this virus, warrant special attention and care. These findings contribute to a better understanding of the cognitive effects associated with COVID-19 and underscore the need for further longitudinal research. Although the pandemic was declared over in May 2023—without implying that the virus has been eradicated—the working conditions of healthcare professionals should continue to be studied to preserve the health of this population.

## Figures and Tables

**Table 1:** Sociodemographic data and symptomatology (only for the COVID-19 group). Means, percentages, and standard deviations (in parentheses) are reported. Statistical details of group comparisons (Student’s *t*-test or χ^2^-tests) are also displayed.

Variables	Percentage or Mean (SD)		Group Effect	
	Control Group	COVID-19 Group	Statistic t or χ^2^	*p*-Value
Age (years)	44.24 (11.23)	45.18 (10.49)	−0.415	0.679
Education			0.393	0.822
*Intermediate studies (%)*	33.3	32.0		
*Advanced vocational qualification (%)*	4.8	8.0		
*University studies (%)*	61.9	60.0		
Profession			7.640	0.054
*Physician (%)*	16.7	6.0		
*Nurse (%)*	54.8	80.0		
*Physiotherapist (%)*	14.3	4.0		
*Other (%)*	14.3	10.0		
Time since COVID-19 symptoms (months)	-	13 (3.82)		
COVID-19 Symptoms				
*Fever (%)*	-	72.0		
*Dry cough (%)*	-	66.0		
*Tiredness (%)*	-	90.0		
*Aches and pains (%)*	-	78.0		
*Sore throat (%)*	-	52.0		
*Diarrhea (%)*	-	32.0		
*Conjunctivitis (%)*	-	6.0		
*Headache (%)*	-	84.0		
*Loss of smell (%)*	-	70.0		
*Loss of taste (%)*	-	64.0		
*Dysgeusia (%)*	-	34.0		
*Skin rash or loss of pain (%)*	-	14.0		
*Difficulty breathing or feeling shortness of breath (%)*	-	48.0		
*Chest pain or pressure (%)*	-	46.0		
*Inability to speak or move (%)*	-	8.0		
*Vomiting or nausea (%)*	-	22.0		
*Itchy skin (%)*	-	12.0		
*Feeling dizzy (%)*	-	32.0		
*Syncope (%)*	-	4.0		
*Confusion (%)*	-	10.0		

**Table 2:** Means and standard deviations (in parentheses) of neuropsychological scores, split by group. Raw scores are presented.

Neuropsychological Tests	COVID-19 Group	Control Group	Statistics (df = 1.90)
***Attention***			
D2 Test			
*Scanning speed*	432.38 (87.494)	434.83 (81.874)	F = 0.018, *p* = 0.895
*Scanning accuracy*	170.10 (43.239)	165.55 (38.952)	F = 0.277, *p* = 0.600
*Omission*	12.52 (10.300)	17.31 (14.146)	F = 3.516, *p* = 0.064
*Commission*	7.90 (4.807)	2.45 (4.915)	F = 1.957, *p* = 0.165
*Effectiveness index*	412.48 (82.240)	406.93 (86.358)	F = 0.099, *p* = 0.753
*Concentration index*	162.18 (37.307)	163.10 (40.975)	F = 0.013, *p* = 0.911
*Max*	36.96 (6.114)	36.76 (5.656)	F = 0.026, *p* = 0.873
*Min*	25.14 (7.709)	24.40 (6.950)	F = 0.227, *p* = 0.635
*Variation index*	12.04 (4.882)	12.36 (5.093)	F = 0.093, *p* = 0.762
TESEN			
*Path 1 execution*	27.01 (7.695)	28.67 (5.549)	F = 1.357, *p* = 0.247
*Path 1 speed*	92.88 (25.749)	86.21 (20.290)	F = 1.849, *p* = 0.177
*Path 1 accuracy*	98.59 (2.840)	98.98 (2.473)	F = 0.499, *p* = 0.482
*Path 2 execution*	25.01 (6.653)	26.56 (6.200)	F = 1.305, *p* = 0.256
*Path 2 speed*	99.46 (25.565)	94.36 (29.597)	F = 0.787, *p* = 0.377
*Path 2 accuracy*	98.33 (3.039)	98.23 (3.553)	F = 0.018, *p* = 0.893
*Path 3 execution*	18.31 (4.567)	18.89 (4.371)	F = 0.388, *p* = 0.535
*Path 3 speed*	106.54 (36.249)	99.81 (25.072)	F = 1.032, *p* = 0.312
*Path 3 accuracy*	95.96 (10.791)	95.69 (7.221)	F = 0.018, *p* = 0.894
*Path 4 execution*	15.88 (19.016)	14.39 (4.271)	F = 0.250, *p* = 0.619
*Path 4 speed*	144.02 (3.759)	139.19 (40.697)	F = 0.027, *p* = 0.555
*Path 4 accuracy*	95.51 (13.201)	98.04 (3.387)	F = 1.468, *p* = 0.229
*Total execution*	19.57 (4.591)	20.94 (4.24)	F = 2.193, *p* = 0.142
*Total speed*	432.81 (128.36)	418.62 (104.04)	F = 0.331, *p* = 0.567
*Total accuracy*	97.54 (4.125)	97.83 (2.846)	F = 0.151, *p* = 0.699
Symbol search	37.25 (8.411)	37.78 (8.568)	F = 0.084, *p* = 0.773
Number Key	75.26 (14.151)	79.36 (17.072)	F = 1.585, *p* = 0.211
***Memory***			
Word List I			
*Score 1 recall*	6.62 (1.398)	6.67 (1.509)	F = 0.024, *p* = 0.878
*Total recall score*	35.80 (5.063)	36.10 (4.674)	F = 0.083, *p* = 0.774
*Interference 1*	0.92 (1.441)	0.81 (2.027)	F = 0.093, *p* = 0.761
*Learning*	3.72 (1.371)	3.95 (1.738)	F = 0.514, *p* = 0.475
*Interference 2*	1.14 (1.552)	1.21 (1.353)	F = 0.059, *p* = 0.809
Word List II			
*Recall*	9.04 (2.466)	9.02 (2.342)	F = 0.001, *p* = 0.974
*Recognition*	23.39 (1.169)	23.17 (1.305)	F = 0.726, *p* = 0.396
Faces I	40.87 (3.512)	40.60 (3.787)	F = 0.128, *p* = 0.721
Faces II	102.65 (15.806)	100.55 (7.765)	F = 0.619, *p* = 0.433
Vocabulary	36.24 (9.317)	34.00 (9.904)	F = 1.246, *p* = 0.267
Rey–Osterrieth Complex Figure			
*Copy*	33.15 (4.136)	32.77 (3.239)	F = 0.229, *p* = 0.633
*Copy time*	125.40 (38.970)	137.52 (43.747)	F = 1.975, *p* = 0.163
*Recovery*	18.06 (5.985)	18.45 (6.288)	F = 0.094, *p* = 0.760
*Recovery time*	109.84 (37.433)	117.20 (43.584)	F = 0.750, *p* = 0.389
***Executive Functions***			
FAS Test			
*F (Words/min)*	18.04 (4.256)	19.60 (3.749)	F = 3.299, *p* = 0.073
*A (Words/min)*	15.76 (4.461)	16.31 (3.646)	F = 0.408, *p* = 0.525
*S (Words/min)*	15.18 (4.308)	15.60 (3.722)	F = 0.240, *p* = 0.626
*Semantics (Words/min)*	63.18 (11.208)	66.93 (10.778)	F = 2.644, *p* = 0.107
Five Digit Test			
*Reading*	20.14 (3.417)	19.74 (3.507)	F = 0.308, *p* = 0.580
*Counting*	21.88 (3.409)	21.19 (3.430)	F = 0.929, *p* = 0.338
*Choosing*	31.96 (5.525)	30.52 (5.438)	F = 1.565, *p* = 0.214
*Shifting*	41.30 (8.812)	39.07 (7.270)	F = 1.709, *p* = 0.194
*Inhibition*	11.82 (5.054)	10.95 (5.860)	F = 0.582, *p* = 0.448
*Flexibility*	21.16 (7.950)	19.26 (7.398)	F = 1.385, *p* = 0.242
Spatial Span			
*Forward*	8.60 (1.841)	8.71 (1.672)	F = 0.096, *p* = 0.758
*Backward*	8.22 (1.962)	7.95 (1.766)	F = 0.465, *p* = 0.497
*Total*	17.00 (3.812)	16.62 (2.946)	F = 0.279, *p* = 0.599
Letter–Number Sequencing	19.12 (2.344)	19.69 (2.552)	F = 1.247, *p* = 0.267
Digit Span			
*Forward*	9.00 (1.959)	8.69 (2.089)	F = 0.536, *p* = 0.466
*Forward Span*	6.06 (1.168)	5.93 (1.237)	F = 0.274, *p* = 0.602
*Backward*	8.28 (2.286)	8.29 (1.798)	F = 0.000, *p* = 0.990
*Backward Span*	4.62 (1.244)	4.67 (1.052)	F = 0.037, *p* = 0.848
*Increasing*	8.46 (2.279)	8,79 (2.280)	F = 0.466, *p* = 0.496
*Increasing Span*	5.84 (1.695)	6.29 (2.351)	F = 1.111, *p* = 0.295
*Total*	25.76 (5.286)	25.76 (4.616)	F = 0.000, *p* = 0.999
Similarities	21.88 (4.914)	19.93 (4.566)	F = 3.838, *p* = 0.053
Key Search	10.80 (3.774)	11.52 (3.487)	F = 0.900, *p* = 0.345
Key Search time	60.42 (61.305)	58.26 (57.274)	F = 0.030, *p* = 0.863
Zoo Test Total	11.10 (3.898)	11.40 (3.964)	F = 0.137, *p* = 0.712

Abbreviations: D2 Sustained Attention Test: D2 Test; Test of the Paths: TESEN.

**Table 3:** Means and standard deviations (in parentheses) of self-reported emotional scores by group. Raw scores are presented.

Emotional Measures	COVID-19 Group	Control Group	Statistics (df = 1.90)
STAI-State	25.08 (4.923)	24.67 (5.664)	F = 0.113, *p* = 0.738
STAI-Trait	26.52 (5.578)	24.07 (5.110)	F = 4.746, *p* = **0.032**
PHQ-9	8.06 (5.482)	7.71 (4.994)	F = 1.748, *p* = 0.190
MBI	11.32 (5.156)	12.05 (6.231)	F = 1.079, *p* = 0.541
PCL	34.58 (11.500)	34.55 (17.429)	F = 0.639, *p* = 0.426
MFE-30	14.42 (8.442)	13.76 (8.678)	F = 1.303, *p* = 0.992
ISP-20	15.72 (11.644)	14.76 (12.689)	F = 0.057, *p* = 0.812
SCL-90-R	78.32 (56.750)	69.50 (64.569)	F = 0.008, *p* = 0.927
*Somatizations*	1.01 (0.783)	1.70 (5.025)	F = 0.245, *p* = 0.622
*Obsessions and compulsions*	1.28 (0.868)	2.45 (10.078)	F = 0.097, *p* = 0.756
*Interpersonal sensitivity*	0.84 (0.773)	0.58 (0.556)	F = 0.909, *p* = 0.343
*Depression*	1.13 (0.881)	0.81 (0.768)	F = 0.822, *p* = 0.367
*Anxiety*	0.69 (0.642)	0.68 (0.782)	F = 0.674, *p* = 0.414
*Hostility*	0.68 (0.705)	0.41 (0.671)	F = 1.433, *p* = 0.235
*Phobic anxiety*	0.27 (0.426)	0.32 (0.535)	F = 1.618, *p* = 0.207
*Paranoid ideation*	0.66 (0.809)	0.37 (0.562)	F = 1.925, *p* = 0.169
*Psychoticism*	0.42 (0.620)	0.26 (0.362)	F = 2.988, *p* = 0.141
*Global severity index*	0.84 (0.647)	0.67 (0.628)	F = 0.176, *p* = 0.676
*Positive discomfort index*	1.66 (0.575)	1.76 (0.612)	F = 3.672, *p* = 0.059
*Total positive symptoms*	41.78 (21.076)	34.02 (23.912)	F = 0.856, *p* = 0.357

Abbreviations: Symptom Checklist-90: SCL-90-R, Abbreviated Prefrontal Symptom Inventory: ISP-20, Failures in Everyday Life Questionnaire: MFE-30, Post-Traumatic Stress Checklist–Civilian Version: PCL, Emotional Exhaustion Subscale of Maslach’s Burnout Inventory: MBI, Patient Health Questionnaire-9: PHQ-9, and State-Trait Anxiety Inventory: STAI.

**Table 4:** Pearson correlations between neuropsychological measures and self-reported emotional scores for the entire sample of participants.

**Emotional Tests**	**STAI State**	**STAI Trait**	**PHQ-9**	**MBI**	**PCL**	**MFE-30**	**ISP20**	**SCL-90-R**	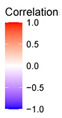
**Neuropsychological tests**									
***Attention***									
D2 Test									
*Scanning speed*	−0.040	−0.023	0.038	−0.105	−0.131	0.037	0.048	−0.086	
*Scanning accuracy*	0.001	0.005	0.028	−0.115	−0.080	0.007	0.009	−0.068	
*Omission*	−0.084	0.015	0.005	−0.034	−0.015	0.101	0.126	−0.010	
*Commission*	0.002	0.066	−0.057	−0.038	0.017	−0.067	−0.044	−0.006	
*Effectiveness index*	0.000	−0.140	0.061	−0.205 *	−0.133	0.009	0.033	−0.103	
*Concentration index*	0.000	−0.027	0.057	−0.104	−0.093	0.040	0.030	−0.069	
*Max*	−0.073	0.058	0.141	−0.104	−0.027	0.113	0.105	0.002	
*Min*	−0.104	−0.050	−0.101	−0.207 *	−0.201 *	−0.137	−0.006	−0.163	
*Variation index*	0.078	0.137	0.328 **	0.199	0.273 **	0.328 **	0.147	0.249 *	
TESEN									
*Path 1 execution*	−0.024	−0.057	0.008	−0.179	−0.088	−0.123	−0.039	−0.125	
*Path 1 speed*	−0.003	0.086	0.067	0.177	0.113	0.110	0.061	0.132	
*Path 1 accuracy*	0.024	0.059	0.092	0.024	0.068	0.013	−0.051	−0.085	
*Path 2 execution*	0.031	−0.152	−0.039	−0.188	−0.209 *	−0.237 *	−0.145	−0.182	
*Path 2 speed*	−0.038	0.125	0.037	0.215 *	0.178	0.176	0.137	0.146	
*Path 2 accuracy*	0.134	−0.112	−0.161	004	−0.001	−0.093	−0.087	−0.064	
*Path 3 execution*	0.027	−0.016	0.134	−0.053	0.053	0.105	0.018	−0.026	
*Path 3 speed*	−0.089	−0.001	−0.091	0.075	−0.049	−0.080	0.004	0.085	
*Path 3 accuracy*	−0.038	−0.008	0.028	0.064	0.074	0.096	0.079	0.079	
*Path 4 execution*	0.100	−0.035	0.189	−0.003	0.151	0.247 *	0.135	0.118	
*Path 4 speed*	0.046	0.204 *	0.039	0.275 **	0.176	0.079	0.169	0.217 *	
*Path 4 accuracy*	0.055	0.050	0.015	−0.013	0.010	−0.031	−0.044	−0.068	
*Total execution*	−0.022	−0.128	0.022	−0.225 *	−0.124	−0.100	−0.104	−0.168	
*Total speed*	−0.013	0.121	0.037	0.228 *	0.133	0.106	0.104	0.183	
*Total accuracy*	0.031	0.006	−0.033	0.032	0.046	−0.007	−0.042	−0.044	
Symbol Search	0.121	−0.001	0.049	−0.053	−0.012	0.005	0.051	0.022	
Number Key	0.079	0.059	−0.048	0.016	−0.138	−0.076	−0.021	−0.056	
***Memory***									
Word List I									
*Score 1 recall*	−0.044	0.030	−0.043	−0.129	−0.077	−0.147	−0.091	−0.167	
*Total recall score*	−0.087	0.143	0.078	0.007	0.003	0.003	0.022	−0.081	
*Interference 1*	−0.130	−0.047	−0.126	−0.175	−0.130	−0.164	−0.100	−0.095	
*Learning*	−0.034	0.126	0.150	0.177	0.064	0.155	0.072	0.097	
*Interference 2*	−0.082	0.078	0.112	0.117	0.192	0.043	0.014	0.112	
Word List II									
*Recall*	−0.010	0.111	0.020	−0.007	−0.096	0.026	−0.026	−0.095	
*Recognition*	0.070	0.141	0.167	0.078	0.067	0.184	0.170	0.101	
Faces I	−0.013	−0.006	0.011	−0.170	−0.112	0.050	−0.005	−0.036	
Faces II	0.031	0.104	0.128	0.130	0.134	0.031	0.060	0.094	
Vocabulary	−0.022	0.048	−0.225 *	−0.149	−0.183	−0.125	−0.091	−0.242 *	
Rey–Osterrieth Complex Figure									
*Copy*	−0.179	−0.016	−0.041	−0.036	−0.161	−0.096	−0.110	−0.178	
*Copy time*	−0.018	0.023	0.026	0.261 *	0.141	0.064	−0.024	0.098	
*Recovery*	−0.161	−0.155	−0.046	−0.217 *	−0.294 *	−0.101	−0.167	−0.261 *	
*Recovery time*	0.039	0.090	−0.035	−0.002	−0.016	−0.101	−0.196	−0.127	
***Executive Functions***									
FAS Test									
*F (Words/min)*	−0.007	−0.016	−0.090	−0.042	−0.049	−0.073	−0.058	−0.063	
*A (Words/min)*	−0.053	−0.104	−0.168	−0.022	−0.087	−0.012	−0.056	−0.094	
*S (Words/min)*	−0.040	−0.092	−0.108	−0.157	−0.114	−0.073	−0.118	−0.170	
*Semantics (words/3min)*	−0.047	−0.098	−0.182	−0.127	−0.217 *	−0.065	−0.093	−0.209 *	
Five Digit Test									
*Reading*	−0.161	0.043	0.159	−0.053	0.138	−0.020	−0.060	0.047	
*Counting*	−0.113	0.071	0.188	−0.021	0.131	−0.029	−0.023	0.074	
*Choosing*	−0.059	0.068	0.067	−0.010	0.065	0.035	0.123	0.063	
*Shifting*	−0.036	−0.053	−0.015	0.056	0.067	0.027	0.049	0.105	
*Inhibition*	0.066	0.064	−0.004	0.067	0.011	0.072	0.177	0.064	
*Flexibility*	0.027	−0.083	−0.096	0.070	−0.001	0.030	0.074	0.081	
Spatial Span									
*Forward*	−0.029	−0.077	0.030	−0.026	−0.020	−0.040	0.017	−0.042	
*Backward*	−0.064	−0.068	−0.027	−0.279 **	−0.167	−0.035	−0.114	−0.263 *	
*Total*	−0.030	−0.039	−0.017	−0.186	−0.109	−0.069	−0.038	−0.179	
Letter–Number Sequencing	0.069	−0.092	0.026	−0.101	−0.074	0.043	−0.053	−0.116	
Digit Span									
*Forward*	0.040	−0.106	−0.156	−0.257 *	−0.104	−0.098	−0.144	−0.198	
*Forward* Span	0.016	−0.124	−0.221 *	−0.279 **	−0.132	−0.132	−0.211 *	−0.235 *	
*Backward*	0.011	−0.111	−0.050	−0.110	−0.055	−0.059	−0.084	−0.102	
*Backward* Span	0.024	−0.97	−0.020	−0.057	−0.047	−0.046	−0.111	−0.095	
*Increasing*	−0.081	−0.274 **	−0.060	−0.159	−0.091	0.010	−0.048	−0.147	
*Increasing* Span	0.035	−0.240 *	−0.004	−0.109	−0.017	0.115	−0.062	−0.074	
*Total*	−0.012	−0.209 *	−0.109	−0.224 *	−0.106	−0.061	−0.116	−0.190	
Similarities	0.071	−0.036	−0.148	−0.063	−0.141	0.007	0.047	−0.096	
Key Search	0.091	0.068	−0.019	0.044	−0.108	0.029	0.070	−0.056	
Key Search Time	−0.154	−0.120	0.046	0.056	0.061	0.044	−0.042	0.021	
Zoo Test Total	0.081	0.177	−0.007	0.163	−0.051	−0.125	0.061	−0.022	

* *p* < 0.05; ** *p* < 0.01. Abbreviations: State-Trait Anxiety Inventory: STAI, Patient Health Questionnaire-9: PHQ-9, Emotional Exhaustion Subscale of Maslach’s Burnout Inventory: MBI, Post-Traumatic Stress Checklist–Civilian Version: PCL, Failures in Everyday Life Questionnaire: MFE-30, Abbreviated Prefrontal Symptom Inventory: ISP-20, Symptom Checklist-90: SCL-90-R, D2 Sustained Attention Test: D2 test, and Test of the Paths: TESEN.

## Data Availability

The data are available at https://osf.io/fd35j/?view_only=27f14638244340e3ae229ca318200944 (accessed on 20 July 2025).

## Abbreviations

The following abbreviations are used in this manuscript:

BADS	Behavioral Assessment of the Dysexecutive Syndrome
COVID-19	Coronavirus Disease 2019
CoVs	Coronavirus
HUFA	University Hospital Alcorcon Foundation
ISP-20	Prefrontal Symptom Inventory
MBI	Maslach’s Burnout Inventory
MFE-30	Failures in Everyday Life Questionnaire
MoCA	Montreal Cognitive Assessment
PCL	Post-Traumatic Stress Checklist–Civilian
PHQ-9	Patient Health Questionnaire-9
SARS-CoV-2	Severe acute respiratory syndrome coronavirus 2
SCL-90	Symptom Checklist-90
STAI	State-Trait Anxiety Inventory
TESEN	The Test of the Paths
TMT	Trail Making Test
WAIS-IV	Wechsler Intelligence Scale for Adults-IV
WMS-III	Wechsler Memory Scale III

## Appendix A

**Table A1:** Summary of the neuropsychological tests and the cognitive functions they assess.

Test/Subtest	Cognitive Function	Description
D2 Sustained Attention Test	Sustained and selective attention, processing speed	This test evaluates the ability to maintain attention and discriminate stimuli and provides concentration and processing speed indices.
TESEN (Test of the Paths, TMT-based)	Switching attention, cognitive flexibility	This test assesses task switching, sequential planning, and processing speed; errors and time in the task indicate flexibility efficiency.
Symbol Searching and Number Key (WAIS-IV)	Processing speed, visuomotor coordination	Processing speed and visual scanning are the abilities used to successfully accomplish these tests; the combined scores of both provide the Processing Speed Index.
Word Lists I and II, Faces I and II (WMS-III)	Verbal and visual memory	These instruments evaluate learning, retention, and recognition of verbal and visual information; they assess immediate and delayed memory.
Vocabulary (WAIS-IV)	Semantic memory, verbal reasoning	The vocabulary test is designed to evaluate verbal comprehension and semantic memory.
Rey–Osterrieth Complex Figure Test	Visuospatial construction, visual memory, planning	Through copying and recalling a complex figure, this test assesses visuospatial organization and memory.
Five Digit Test	Executive functions, inhibition, flexibility	This instrument measures inhibition and cognitive flexibility through four subtests: reading, counting, selection, and switching. It provides two indices: inhibition and flexibility.
Digit Span, Spatial Span, Letter–Number Sequencing (WMS-III)	Working memory	This test assesses verbal and visuospatial working memory through the ability to maintain and manipulate information.
Similarities (WAIS-IV)	Verbal reasoning, abstraction	This test assesses reasoning by comparing concepts and forming abstract judgments.
FAS Test	Verbal fluency	This test measures semantic word retrieval, spontaneous flexibility, and semantic memory organization.
Key Search and Zoo (BADS)	Planning, problem-solving, executive functions	This instrument evaluates successful strategies to find lost keys inside a closed space, presented as a square on a sheet of paper, as an evaluation of planning, behavioral monitoring, and problem-solving.
